# Resting-state functional connectivity of the default mode network as a predictor for escitalopram response in adolescents with depression

**DOI:** 10.1038/s41386-026-02442-x

**Published:** 2026-05-21

**Authors:** Moonyoung Jang, Kyung Hwa Lee, Jiyoon Shin, Jung Lee, Jae Hyun Yoo, Jeeyoung Chun, Jisung Ahn, Jae-Won Kim

**Affiliations:** 1 Department of Psychiatry, Korea University Guro Hospital, Seoul, South Korea; 2 Department of Psychiatry, Seoul National University Hospital and Seoul National University College of Medicine, Seoul, South Korea; 3 Department of Psychiatry, Boramae Medical Center, Seoul, South Korea; 4 Integrative Care Hub, Seoul National University Children’s Hospital, Seoul, South Korea; 5 Department of Psychiatry, The Catholic University of Korea, Seoul St Mary’s Hospital, Seoul, South Korea

**Keywords:** Predictive markers, Depression

## Abstract

Adolescent depression differs from adult depression in neurobiology and treatment response. Although resting-state functional connectivity (rsFC) of the default mode network (DMN) has been linked to treatment response in adults, its value as a biomarker in adolescents remains unclear. This study investigated whether baseline DMN rsFC predicts response to selective serotonin reuptake inhibitor (SSRI) treatment in adolescents with major depressive disorder (MDD). Seventy medication-naïve adolescents with MDD (ages 12-17 years) underwent baseline resting-state functional magnetic resonance imaging and an 8-week open-label escitalopram trial. Depressive symptoms were measured using the Children’s Depression Rating Scale-Revised (CDRS-R). Seed-based DMN rsFC analyses used the ventromedial prefrontal cortex (vMPFC), dorsomedial prefrontal cortex (dMPFC), and posterior cingulate cortex (PCC) as seeds. Voxel-wise regressions assessed associations between baseline rsFC and treatment response, defined as the change in CDRS-R score from baseline to week 8. Our results showed that greater baseline DMN rsFC consistently predicted SSRI treatment response in adolescents with MDD. Stronger vMPFC-postcentral gyrus, vMPFC-insula, dMPFC-supramarginal, and PCC-supramarginal gyrus rsFCs at baseline were significantly associated with greater treatment response, as reflected by greater reductions in CDRS-R score following escitalopram treatment (*r* = -0.498 to -0.603, all *p*_*s*_ < 0.001). However, rsFCs within the DMN did not predict SSRI treatment response (all *p*_*s*_ > 0.05). These findings suggest that baseline rsFC between DMN hubs and regions in the sensorimotor and frontoparietal networks may serve as a biomarker of SSRI treatment response in adolescents with depression. This could support individualized treatment strategies within precision psychiatry for youth with MDD.

## Introduction

Adolescent depression is associated with distinct pathophysiological mechanisms compared to adult depression, and prognostic predictors are likewise believed to differ between these groups [1, 2]. Neurobiological distinctions in adolescent depression include differences in serotonin and dopamine receptor distribution [3], as well as altered functional connectivity (FC) within the default mode and limbic networks [4–7]. While treatment responsiveness has been more thoroughly studied in adults, data on adolescent populations remain limited [8, 9]. Early prediction of treatment response in adolescent depression is critically important, as persistent symptoms during this developmental period can lead to long-term consequences, including impaired academic and social functioning and increased risk for psychiatric morbidity in adulthood [10–12].

Selective serotonin reuptake inhibitors (SSRIs) are the first-line pharmacologic treatment for adolescent depression [13]. However, preclinical studies have shown that juvenile animals exposed to SSRIs exhibit increased serotonin receptor expression, which may paradoxically reduce synaptic serotonin availability [14, 15]. This mechanism has been proposed to contribute to clinical phenomena such as SSRI resistance and activation syndrome in adolescents [16, 17]. Early identification of individuals unlikely to respond to SSRIs may help facilitate a timely transition to alternative therapeutic strategies [18, 19].

Despite extensive efforts to identify predictors of SSRI treatment response in adolescent depression, including clinical, familial, and neurocognitive factors, no biomarker has demonstrated sufficient predictive value for integration into clinical guidelines [1, 13, 18]. A recent large-scale meta-analysis in adults with depression reported that neuroimaging markers, particularly resting-state functional MRI (rsfMRI), outperformed clinical variables in predicting treatment outcomes across diverse interventions [9]. Concerns regarding the reliability of fMRI biomarkers have primarily focused on task-evoked activation contrasts [20], whereas resting-state functional connectivity has generally shown more stable test-retest reliability and has been widely used in individual-differences research [21, 22]. Among rsfMRI indices, FC within the default mode network (DMN) showed the highest predictive utility for treatment response [9]. Notably, stronger DMN rsFC has been associated with better SSRI outcomes in adults [23]. Specifically, increased connectivity between the DMN and regions in the frontoparietal network (FPN), such as the angular gyrus, has also been linked to a favorable response in prior studies [23–25]. These findings suggest that DMN rsFC may serve as a candidate biomarker for predicting SSRI treatment response [18].

The DMN comprises three core hubs: the ventromedial prefrontal cortex (vMPFC), dorsomedial prefrontal cortex (dMPFC), and posterior cingulate cortex (PCC) [26]. It plays a central role in self-referential thinking and internally directed cognition, domains implicated in depressive rumination and maladaptive self-focus [27]. These characteristics may explain why DMN rsFC has emerged as a strong predictor of treatment response in adults with depression [28]. However, studies evaluating the predictive value of DMN connectivity in adolescents remain limited [6, 7]. One previous study in adolescents found that reduced DMN rsFC was associated with a favorable SSRI response [29]. To our knowledge, no rsfMRI study to date has examined SSRI response in adolescents with MDD using the vMPFC, dMPFC, or PCC as seed regions. Thus, evaluating DMN rsFC as a biomarker of treatment response in adolescent depression remains a necessary and understudied research direction.

Accordingly, the present study aimed to examine whether the rsFC of the DMN can predict treatment response in adolescents with depression. Baseline depressive symptoms and rsfMRI data were collected before treatment initiation. All participants then underwent an 8-week course of SSRI therapy, followed by a post-treatment assessment of depressive symptoms. Based on prior research [26], we selected the vMPFC, dMPFC and PCC as seed regions within the DMN. We then evaluated whether baseline rsFC in these regions could predict symptom change following SSRI treatment. First, based on evidence from both adult and adolescent populations [23, 29], we hypothesized that altered rsFC within the DMN would predict a favorable response to SSRI treatment. Second, given prior findings that increased rsFC between the DMN and regions within the FPN is associated with better SSRI outcomes [23–25], we hypothesized that higher baseline DMN-FPN connectivity would predict improved treatment response.

## Methods

### Participants

A total of 95 adolescents with MDD were included in the study. Inclusion criteria consisted of adolescents aged 12 to 17 years who met the Diagnostic and Statistical Manual of Mental Disorders, Fifth Edition (DSM-5) [30] criteria for MDD. Diagnosis was established using the Kiddie Schedule for Affective Disorders and Schizophrenia for School-Age Children-Present and Lifetime Version (K-SADS-PL) [31], administered by trained clinical psychologists. Diagnostic interviews were conducted by trained psychologists who achieved high inter-rater reliability (κ ≥ 0.80). All diagnoses were confirmed by a board-certified child and adolescent psychiatrist (J.-W.K.). Participants were recruited from inpatient and outpatient services at the Department of Child and Adolescent Psychiatry, Seoul National University Hospital. Additional details regarding our participants’ diagnostic assessment and recruitment procedures are provided in the Supplementary Methods.

Exclusion criteria were: (a) chronic medical conditions; (b) a history of psychotic disorders, including schizophrenia or bipolar disorder; (c) a history of eating disorders; (d) developmental disorders such as autism spectrum disorder; (e) alcohol or other substance use disorder within the past 6 months; (f) neurological or physical illness; (g) a first-degree relative with bipolar I disorder; and (h) current or prior use of psychiatric medications. The detailed rationale for specific exclusion criteria, including the exclusion of individuals at high familial risk for bipolar disorder, is described in the Supplementary Methods.

All adolescents included in the study were medication-naïve, and approximately 80% were experiencing their first depressive episode at the time of enrollment, whereas the remaining participants had a history of at least one prior depressive episode. The prevalence of comorbid diagnoses and suicidality at baseline is summarized in Supplementary Table 1.

All diagnostic assessments were completed prior to MRI data acquisition, which was conducted before the initiation of SSRI treatment. Of the 95 adolescents initially enrolled, participants were subsequently excluded due to excessive head motion, imaging artifacts, or anatomical abnormalities identified during preprocessing (Supplementary Fig. 1). The demographic and clinical characteristics of the final analytic sample included in the imaging analyses are summarized in Table 1. Correlations between demographic variables and clinical measures are presented in Supplementary Table 2.

**Table 1:** Clinical and demographic variables of the study participants.

	Mean ± SD	*N* (%)
Age, years	15.06 ± 1.52	
Female		49 (70.0%)
Ethnicity (Korean)		70 (100.0%)
Intelligence quotient	104.6 ± 14.18	
Children’s Depression Rating Scale-Revised score		
Baseline (week 0)	58.59 ± 11.23	
After treatment (week 8)	43.11 ± 12.38	
Change (week 8-week 0)	-15.47 ± 13.81	
Anxiety disorder comorbidity		32 (45.7%)
Daily dose of escitalopram, mg	13.65 ± 3.20	
Head motion	0.062 ± 0.015	

Data are presented as mean ± standard deviation (SD) for continuous variables and as number (percentage) for categorical variables. Change (week 8-week 0) represents the difference in Children’s Depression Rating Scale-Revised (CDRS-R) scores between baseline and week 8. Head motion was calculated according to the Euclidean distance between the 6 rigid-body head motion parameters for 2 continuous time points.

The study was approved by the Institutional Review Board at Seoul National University Hospital (IRB no. 1504-113-668). Written informed consent was obtained from both participants and their legal guardians following a full explanation of study procedures. This study adhered to the Strengthening the Reporting of Observational Studies in Epidemiology (STROBE) guidelines for cohort studies.

### SSRI treatment procedures

All adolescents with MDD participated in an 8-week open-label trial of escitalopram [32, 33]. The initial dosage was 5 mg/day during the first week, increased to 10 mg/day beginning in the second week. Dosage adjustments were made at weeks 4 and 6 based on clinical judgment. Escitalopram could be increased in 5 or 10 mg increments or decreased in 5 mg decrements, with a maximum allowable dose of 30 mg/day. Final dosing was individualized to achieve therapeutic efficacy, as determined by the investigator (J.-W.K.) based on symptom improvement and adverse effect monitoring. Once an optimal dose was established, it was maintained through week 8. The final escitalopram dose maintained through week 8 (or at early discontinuation) was used for descriptive analyses in Supplementary Table 3. Concurrent psychotherapy, including cognitive behavioral therapy, was not permitted during the trial. Treatment adherence was assessed through pill counts [34]. Participants were withdrawn from the study if they were noncompliant, defined as consuming less than 60% of prescribed doses at two consecutive visits.

### Depressive symptom assessment

The Children’s Depression Rating Scale-Revised (CDRS-R) used to assess depressive symptom severity in this study is a clinician-rated instrument widely used and validated for assessing depressive symptom severity in children and adolescents [35]. Interrater reliability was high, with an intra-class correlation coefficient of 0.96 between two independent raters. Adolescents attended clinical visits at weeks 1, 2, 4, 6, and 8 during SSRI treatment, and the CDRS-R was administered at each visit. The primary outcome was treatment response, defined as the change in CDRS-R score from baseline (week 0) to either week 8 or the point of early study discontinuation. Greater reductions in CDRS-R score indicated greater treatment response. Among participants included in the final analytic sample (*n* = 70), five participants discontinued the study prior to week 8 but had usable baseline MRI data and at least one post-baseline clinical assessment. Specifically, two participants discontinued after the week-2 visit, two after the week-4 visit, and one after the week-6 visit. All early discontinuations were due to loss to follow-up. For these participants, treatment response was calculated using the last available CDRS-R score obtained prior to discontinuation, consistent with the predefined outcome definition. Additional details regarding early discontinuation procedures are provided in the Supplementary Methods. Adjusted CDRS-R total scores were used for all statistical analyses [36].

### Resting-state functional MRI (rsfMRI) acquisition

rsfMRI data were acquired using a 3-T Siemens Tim Trio scanner (Siemens Healthineers) equipped with a 12-channel birdcage head coil. Functional images were collected using an interleaved T2*-weighted echo planar imaging sequence with the following parameters: repetition time (TR) = 3000 ms, echo time (TE) = 40 ms, flip angle = 90°, slice thickness = 4.0 mm, in-plane resolution = 3.4 × 3.4 mm, no inter-slice gap, 35 axial slices, and a field of view of 240 mm. The rsfMRI scan lasted approximately 9.7 min, yielding a total of 190 volumes. During the scan, participants were instructed to keep their eyes closed, stay awake, relax, and avoid engaging in specific thoughts.

### rsfMRI preprocessing

rsfMRI data were preprocessed using FreeSurfer version 6.0 (Harvard University) and AFNI (Analysis of Functional NeuroImages) version 21.3.09 (National Institute of Mental Health). The preprocessing pipeline was implemented using AFNI’s afni_proc.py script (https://afni.nimh.nih.gov/pub/dist/doc/program_help/afni_proc.py.html) and applied to the functional images acquired during the 9.7-minute resting-state scan.

Preprocessing included multiple steps: removal of initial volumes, spike correction, slice timing correction, motion correction, co-registration, normalization, and smoothing. Specifically, the first four functional volumes were discarded to allow signal stabilization, and spikes were removed from the remaining volumes. Motion correction, co-registration, and normalization were performed sequentially. Functional images were first aligned to a base volume with the fewest outlier voxels. These volumes were then co-registered to the participant’s T1-weighted structural image, which was nonlinearly warped to standard space (AFNI’s MNI152_T1_2009c template). The same transformation was applied to the functional data. The normalized images were then spatially smoothed using a Gaussian kernel with a 4-mm full-width at half-maximum.

To further reduce noise, the fast ANATICOR method was used to remove anatomy-based and motion-related confounds. Ventricular and white matter signals were regressed out using masks segmented via FreeSurfer; these masks were eroded to reduce gray matter contamination. Cerebrospinal fluid (CSF) and white matter masks were generated using FreeSurfer segmentation of each participant’s anatomical image and subsequently eroded to minimize partial volume effects before being used as nuisance regressors. Additionally, six rigid-body motion parameters were included as nuisance regressors. Volumes with head motion exceeding 0.2 mm (based on Euclidean distance from motion parameters) or containing more than 10% outlier voxels were censored. Participants were excluded if more than 30% of volumes exceeded this motion threshold. A bandpass filter (0.01-0.1 Hz) was applied to retain relevant resting-state frequencies while eliminating low-frequency drift and high-frequency noise. The resulting denoised residual time series was used to compute FC across brain regions.

### Subject-level analyses

Based on prior findings, we hypothesized that functional connectivity within regions of the DMN would be associated with treatment response. For analysis, seed regions within the DMN were selected: vMPFC (MNI coordinates: *x* = 0, *y* = 26, *z* = -18) (Supplementary Fig. 2a), dMPFC (*x* = 0, *y* = 52, *z* = 26) (Supplementary Fig. 2b), and PCC (*x* = -8, *y* = -56, *z* = 26) (Supplementary Fig. 2c). These coordinates were derived from a previous meta-analysis of the DMN [37], and 10-mm radius spherical masks were created around each coordinate for FC analyses.

The mean time series was extracted from each region of interest (ROI), defined by the DMN seed masks. These time series were used to compute ROI-to-ROI functional connectivity between the three DMN seeds, representing within-DMN connectivity. In addition, the seed time series were used to generate seed-to-voxel, whole-brain FC maps for each participant, representing connectivity between DMN seeds and other brain regions. Correlation coefficients (*r* values) were transformed into *z*-scores using Fisher’s *r*-to-*z* transformation. These subject-level connectivity measures were then used for group-level statistical analyses.

### Analysis plan

To test our study hypotheses, group-level regression analyses were conducted using subject-level connectivity measures derived from the DMN seeds. ROI-to-ROI connectivity between DMN seeds was used to test whether within-DMN connectivity predicted treatment response (Hypothesis 1). Voxel-wise seed-to-whole-brain regression analyses were used to test whether baseline connectivity between DMN seeds and other brain regions predicted treatment response (Hypothesis 2).

### Group-level analyses

To test the first hypothesis, we examined whether rsFC within the DMN predicted changes in depressive symptoms by performing regression analyses using the z-score correlation coefficients between the three seeds. To test the second hypothesis, voxel-wise regression analyses were performed using AFNI’s 3dRegAna to evaluate whether baseline seed-to-voxel, whole-brain connectivity of each DMN seed was associated with changes in depressive symptoms, defined as the difference in CDRS-R scores between baseline and the final available assessment (week 8 or the point of early discontinuation). Age and sex were included as covariates. Multiple comparisons were corrected using AFNI’s 3dClustSim, with spatial smoothing estimates calculated via the “acf” method in 3dFWHMx (AFNI version 20.03.01; https://afni.nimh.nih.gov) [38]. Cluster-level significance thresholds were estimated through 10,000 Monte Carlo simulations using a two-sided statistical threshold and second-nearest-neighbor (NN2) clustering. A voxel-wise threshold of *p* < 0.005 was applied, with a family-wise error (FWE)-corrected alpha level of *p* < 0.017, reflecting Bonferroni correction for the three DMN seeds (0.05/3). Clusters exceeding the extent thresholds were considered statistically significant and used to identify regions where baseline connectivity with each DMN seed was significantly associated with treatment-related changes in depressive symptoms. All analyses were conducted between November 2024 and March 2025. The regression analysis was replicated using IBM SPSS Statistics version 29.0.1.0 (IBM Corp., Armonk, N.Y., USA) based on the derived z-scores, and a scatter plot was generated to visualize the relationship.

### Sensitivity and additional analyses

Several sensitivity and additional analyses were conducted to evaluate the robustness of the main findings, including models using percent symptom change, controlling for anxiety comorbidity and head motion. rsfMRI was reanalyzed to examine whether applying global signal regression and using an adolescent-specific template changed the findings. Additional analyses examined the associations between baseline rsFC and escitalopram dose, as well as the potential influence of illness recurrence. Detailed methods are provided in the Supplementary Methods.

## Results

### Demographic and clinical characteristics

Of the 95 adolescents with MDD initially recruited, 2 withdrew their consent after screening, 1 improved depressive symptoms after screening, four were lost to follow-up, and 18 were excluded due to either anatomical abnormalities on MRI or excessive imaging artifacts. Therefore, 70 participants were included in the final analysis. The flow of participants through the study is illustrated in Supplementary Fig. 1.

Participants included in the imaging analyses did not differ from excluded participants on key demographic or clinical variables, although excluded participants were slightly younger (Supplementary Table 4). The mean percentage of censored frames was low (2.52% ± 3.46) and was not associated with clinical variables, with only a modest association observed with sex (*p* = 0.044) (Supplementary Table 5).

### Prediction of treatment response to escitalopram using baseline DMN rsFC

This study evaluated whether baseline DMN rsFC could predict response to escitalopram over 8 weeks. Participants’ age, sex, IQ, CDRS-R scores (baseline, post-treatment, and change), and daily escitalopram dose are summarized in Table 1. Categorical treatment response rates based on conventional CDRS-R criteria [36] are presented in Supplementary Table 6. The trajectory of depressive symptom change across treatment visits is illustrated in Supplementary Fig. 3, which shows the mean CDRS-R scores at each clinical assessment with standard error bars.

#### rsFC within the DMN

Baseline rsFCs among DMN seeds were not associated with changes in CDRS-R scores after treatment (*ps* > 0.05 after Bonferroni correction).

#### rsFC between DMN seeds and brain regions outside the DMN

##### vMPFC seed

**Fig. 1:**
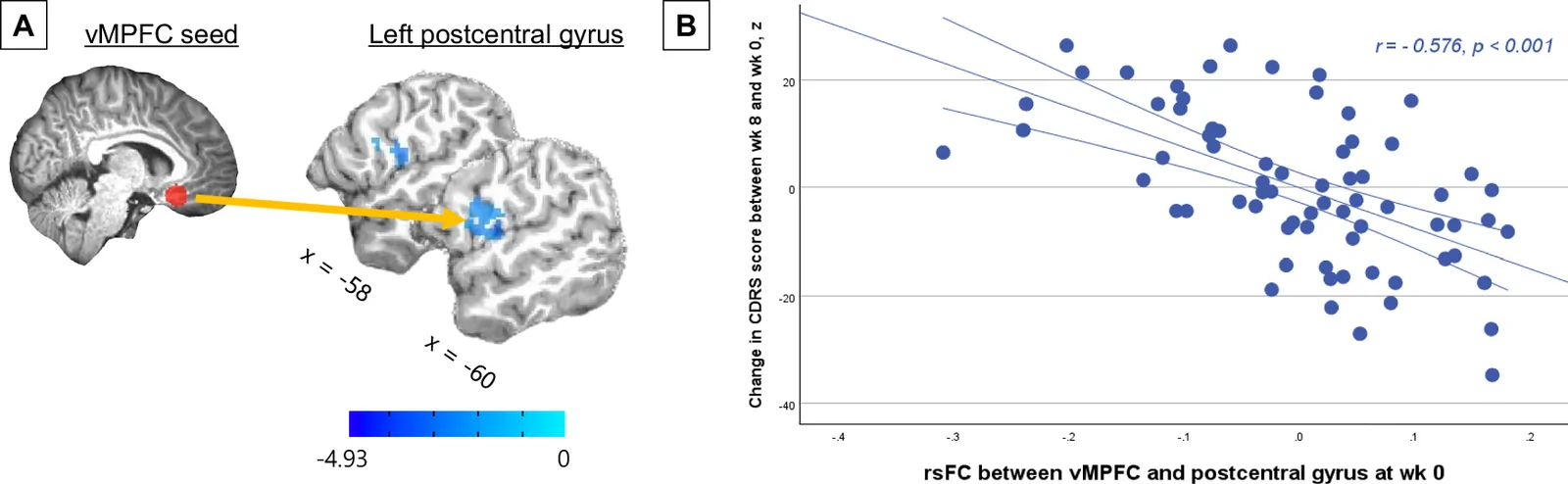
Baseline functional connectivity between the vMPFC and the left postcentral gyrus predicts treatment response. **A** Brain map showing the vMPFC seed (red) and a baseline cluster in the left postcentral gyrus (blue; Brodmann areas 1, 2, 3) exhibiting significant connectivity with the vMPFC. The cluster surpassed the corrected significance threshold (184 voxels; voxel-wise *p* < 0.005, cluster-level *α* = 0.017), with a peak at MNI coordinates at *x* = -57, *y* = -11, *z* = 19. **B** Scatterplot showing that stronger baseline rsFC between the vMPFC and left postcentral gyrus was associated with greater reductions in Children’s Depression Rating Scale-Revised (CDRS-R) scores, as indicated by more negative change values. These reductions reflect greater treatment response after 8 weeks of escitalopram treatment. (*r* = -0.576, *p* < 0.001).

**Fig. 2:**
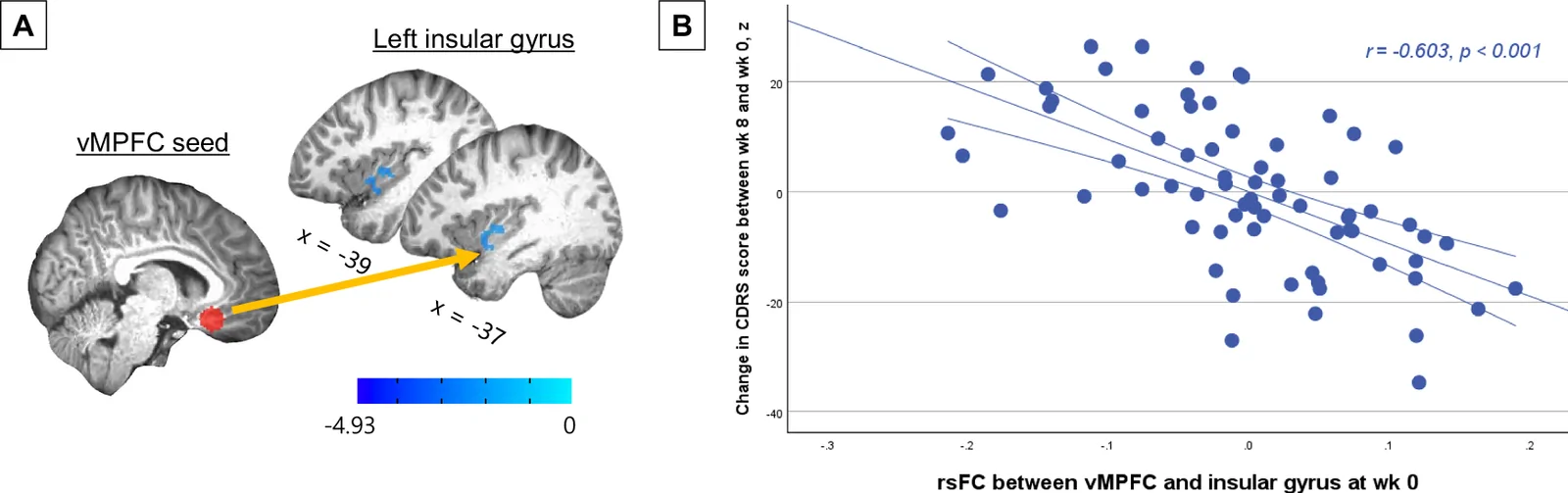
Baseline functional connectivity between the vMPFC and the left insula predicts treatment response. **A** Brain map showing the vMPFC seed (red) and a baseline cluster in the left insula (blue; Brodmann area 13) exhibiting significant connectivity with the vMPFC. The cluster surpassed the corrected significance threshold (184 voxels; voxel-wise *p* < 0.005, cluster-level *α* = 0.017), with a peak at MNI coordinates at *x* = -41, *y* = -11, *z* = -4. **B** Scatterplot showing that stronger baseline rsFC between the vMPFC and left insula was associated with greater reductions in CDRS-R scores, as indicated by more negative change values. These reductions reflect greater treatment response after 8 weeks of escitalopram treatment. (*r* = -0.603, *p* < 0.001).

Regression analysis showed that baseline rsFCs between the vMPFC and the left postcentral gyrus (Brodmann areas 1, 2, 3), and between the vMPFC and the left posterior insula (Brodmann area 13) significantly predicted changes in CDRS-R scores following 8 weeks of escitalopram treatment. Both clusters exceeded the corrected minimum cluster size threshold for the vMPFC seed (184 voxels; voxel-wise threshold *p* < 0.005, cluster-level *α* = 0.017). Figure 1a shows the left postcentral gyrus region (peak MNI coordinate: *x* = -57, *y* = -11, *z* = 19) that exhibited significant baseline rsFC with the vMPFC. Stronger baseline rsFC between these regions was associated with greater reductions in CDRS-R scores after treatment (*r* = -0.576, *p* < 0.001; Fig. 1b). Figure 2a depicts the left insula cluster (peak MNI coordinate: *x* = -41, *y* = -11, *z* = -4) showing significant baseline connectivity with the vMPFC. Stronger baseline rsFC between the vMPFC and the left insula was similarly associated with greater reductions in CDRS-R scores after treatment (*r* = -0.603, *p* < 0.001; Fig. 2b).

##### dMPFC seed

**Fig. 3:**
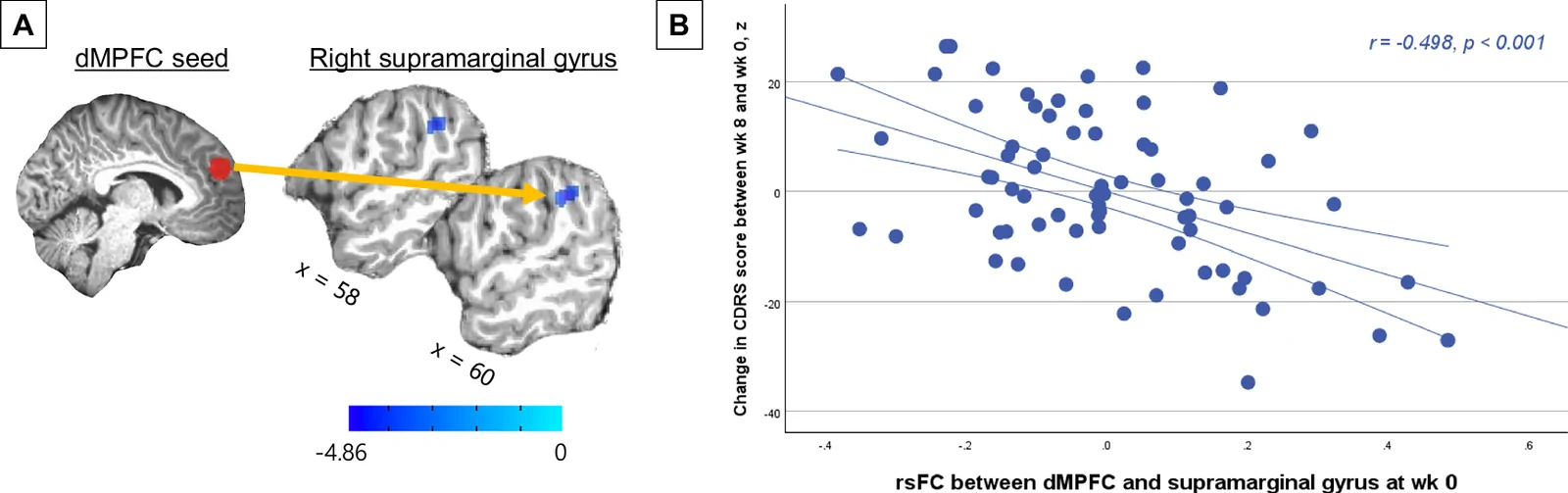
Baseline functional connectivity between the dorsomedial prefrontal cortex (dMPFC) and the right supramarginal gyrus predicts treatment response. **A** Brain map showing the dMPFC seed (red) and a functionally connected cluster in the right supramarginal gyrus (blue; Brodmann area 40) at baseline. This met the corrected significance threshold (177 voxels; voxel-wise *p* < 0.005, cluster-level *α* = 0.017), with its peak at MNI coordinates *x* = 60, *y* = -39, *z* = 36. **B** Scatterplot showing that stronger baseline rsFC between the dMPFC and right supramarginal gyrus was associated with greater reductions in CDRS-R scores, as reflected by more negative change values, indicating treatment response after 8 weeks of escitalopram treatment (*r* = -0.498, *p* < 0.001).

Baseline rsFC between the dMPFC and the right supramarginal gyrus (Brodmann area 40) significantly predicted changes in CDRS-R scores after 8 weeks of escitalopram treatment. The identified cluster exceeded the cluster-extent threshold corrected for multiple comparisons for the dMPFC seed (177 voxels; voxel-wise threshold *p* < 0.005, cluster-level *α* = 0.017; peak MNI coordinate: *x* = 60, *y* = -39, *z* = 36) (Fig. 3a). Notably, individuals with higher baseline rsFC between these regions had greater reductions in CDRS-R scores post-treatment (*r* = -0.498, *p* < 0.001; Fig. 3b).

##### PCC seed

**Fig. 4:**
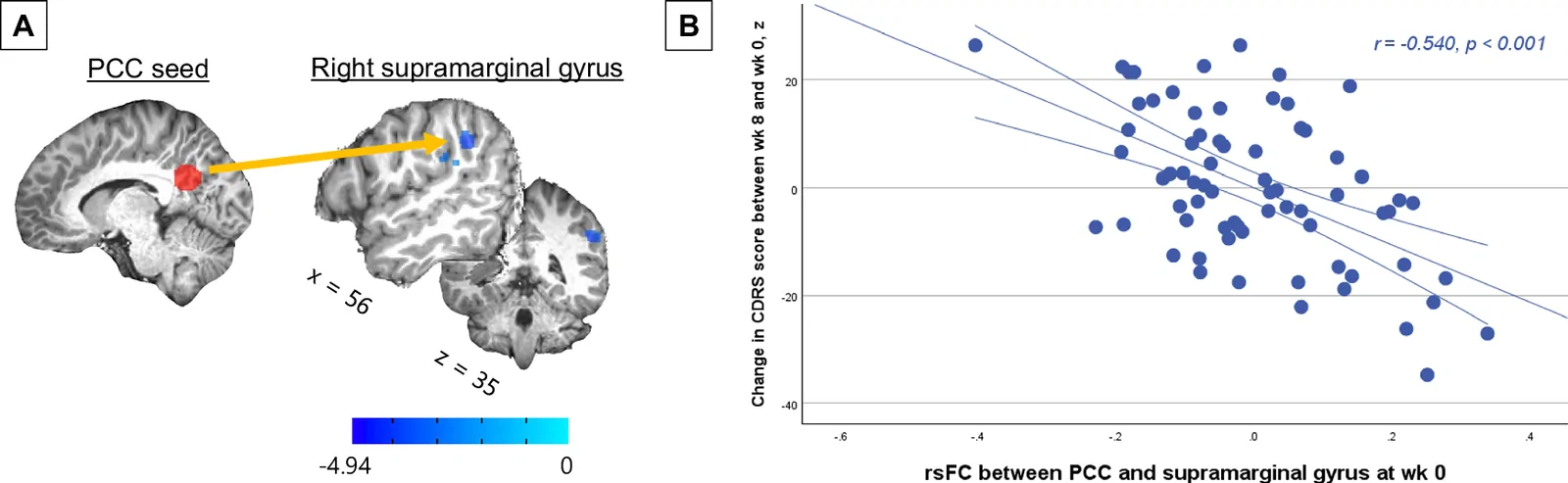
Baseline functional connectivity between the posterior cingulate cortex (PCC) and the right supramarginal gyrus predicts treatment response. **A** Brain map showing the PCC seed (red) and a functionally connected cluster in the right supramarginal gyrus (blue; Brodmann area 40) at baseline. This met the corrected significance threshold (221 voxels; voxel-wise *p* < 0.005, cluster-level *α* = 0.017), with its peak at MNI coordinates *x* = 55, *y* = -43, *z* = 35. **B** Scatterplot showing that stronger baseline rsFC between the PCC and right supramarginal gyrus was associated with greater reductions in CDRS-R scores, as reflected by more negative change values, indicating treatment response after 8 weeks of escitalopram treatment (*r* = -0.540, *p* < 0.001).

Similar to the dMPFC seed result, baseline rsFC between the PCC and the right supramarginal gyrus significantly predicted changes in CDRS-R scores following 8 weeks of escitalopram treatment. This right supramarginal cluster (Brodmann area 40) met the corrected cluster size threshold for the PCC seed (221 voxels; voxel-wise threshold *p* < 0.005, cluster-level *α* = 0.017; peak MNI coordinate: *x* = 55, *y* = -43, *z* = 35) (Fig. 4a). Stronger baseline rsFC between these regions was associated with greater reductions in CDRS-R scores post-treatment (*r* = -0.540, *p* < 0.001; Fig. 4b).

### Sensitivity and additional analyses

#### Associations between baseline rsFC and escitalopram dose

Additional analyses examined whether baseline rsFC measures were associated with the final escitalopram dose. No significant associations were observed between escitalopram dose and any baseline rsFC measure after adjusting for age and sex (all *p*_*s*_ > 0.08; Supplementary Table 7). In addition, the final escitalopram dose was not significantly associated with treatment response, as indexed by change in CDRS-R score, after adjusting for age and sex (*β* = -0.368, *p* = 0.213).

#### Sensitivity analyses addressing baseline symptom severity

When percent symptom change was used as the dependent variable, vMPFC-insula, vMPFC-postcentral, PCC-supramarginal, and dMPFC-supramarginal rsFC each significantly predicted improvement (all *p*_*s*_ < 0.001). The magnitude and direction of effects were highly consistent with the absolute change models. Similarly, when baseline CDRS-R score was included as a covariate, all four rsFC predictors remained significantly associated with treatment response (all *p*_*s*_ < 0.001). Detailed results for both sets of analyses are presented in Supplementary Table 8.

#### Sensitivity analysis controlling for anxiety comorbidity

Including anxiety disorder comorbidity as a covariate did not alter the results; all significant rsFC predictors remained significant. Detailed results are presented in Supplementary Table 9.

#### Episode recurrence-related analysis

There were no significant differences in treatment response between participants with a first depressive episode and those with recurrent episodes, as indexed by CDRS-R change (*p* = 0.88) or percent symptom change (*p* = 0.61). Baseline rsFC values of the significant clusters also did not differ between groups (all *p*_*s*_ > 0.11).

#### Sensitivity analysis controlling for head motion

Including mean head motion as a covariate did not alter the results; all significant rsFC predictors remained significant. Detailed results are presented in Supplementary Table 10.

#### Sensitivity analysis using global signal regression (GSR)

Analyses using GSR-preprocessed data yielded consistent results, with all four connectivity pairs (vMPFC-postcentral, vMPFC-insula, dMPFC-supramarginal, and PCC-supramarginal) remaining significantly associated with treatment response after controlling for age and sex. Although effect sizes were modestly attenuated compared to the results without GSR application, the direction and statistical significance of the associations were preserved. Detailed results are presented in Supplementary Table 11 and Supplementary Fig. 4.

#### Sensitivity analysis using an adolescent-specific template

Reanalysis using an adolescent-specific template (MNIPediatricAsym, cohort 6) [39] yielded clusters in anatomically similar regions to the primary analysis, although with reduced cluster extent. Significant clusters were identified in the left postcentral gyrus (114 voxels, peak MNI: [-63, -9, 12]) and left posterior insula (53 voxels, peak MNI: [-40, -4, 14]) for the vMPFC seed, and in the right supramarginal gyrus for the dMPFC (80 voxels, peak MNI: [61, -44, 37]) and PCC seeds (244 voxels, peak MNI: [59, -39, 30]). rsFC values extracted from these clusters remained significantly associated with reductions in CDRS-R scores after controlling for age and sex. Detailed cluster maps and corresponding scatter plots are presented in Supplementary Figs. 5–8.

## Discussion

This study is the first to investigate whether rsFC of the DMN can predict SSRI treatment response in adolescents with MDD. Baseline rsFC within DMN seeds was not associated with treatment response. In contrast, consistent with our second hypothesis, stronger baseline connectivity between core DMN regions and regions outside the DMN predicted greater symptom improvement following SSRI treatment. Specifically, higher rsFCs between the vMPFC and both the postcentral gyrus and left insula, as well as between the dMPFC and the supramarginal gyrus, and between the PCC and the supramarginal gyrus, were associated with greater reductions in CDRS-R scores. Notably, the supramarginal gyrus is a core node of the FPN, consistent with our hypothesis regarding DMN-FPN connectivity [40].

As hypothesized, rsFCs of key DMN regions were found to predict treatment response. Specifically, stronger baseline rsFCs between the vMPFC and both the postcentral gyrus and the posterior insula predicted greater treatment response. The postcentral gyrus and the posterior insula are both generally classified as part of the somatosensory network (SMN) [41]. In particular, the postcentral gyrus is predominantly responsible for somatosensory processing [42], and the posterior insula acts as a hub integrating sensory inputs [43]. Previous research has shown that lower rsFC between the SMN, which includes the postcentral gyrus, and the DMN is associated with higher levels of rumination, suggesting that greater rsFC in this region may help disengage from internally focused self-referential thinking [27, 44]. In addition, a study in adolescents with depression reported that reduced rsFC between the SMN, which includes both the postcentral gyrus and posterior insula, and the DMN was associated with a greater severity of depressive symptoms. This decrease in connectivity was interpreted as reflecting impaired integration between self-referential processing and external sensory information [45]. Therefore, based on previous findings, it can be inferred that greater baseline rsFC between the postcentral gyrus and the DMN, as observed in the present study, may prevent excessive engagement in self-referential processing and ultimately lead to a greater treatment response to escitalopram.

However, our result that rsFCs between the DMN and SMN regions emerged as potential predictors of treatment response in adolescent depression differs from prior adult studies where predictive markers were primarily limited to DMN-FPN connectivity [23–25]. One possible reason for this difference may be related to the ongoing maturation of cognitive functions during adolescence [46]. In other words, DMN-FPN rsFC may not yet be sufficiently developed to emerge as a predictor of treatment response [47, 48]. Another possibility concerns age-related differences in symptom presentation, with somatic symptoms being relatively more common in youth. A recent study demonstrated that increased DMN-SMN connectivity is associated with maladaptive interpretation of somatic sensations, which, in turn, is linked to the manifestation of somatic symptoms [49]. Such somatic symptoms are known to be more prominent in childhood and adolescent depression than in adult depression [46]. Accordingly, it can be speculated that mechanisms underlying somatic symptoms may constitute a relatively greater proportion of the overall pathophysiology in adolescent depression than in adult cases, and that improvements in these mechanisms may therefore be more closely associated with treatment outcomes in this population. Nevertheless, this interpretation remains hypothetical and warrants further investigation.

Second, higher baseline rsFCs between the dMPFC and the supramarginal gyrus, and between the PCC and the supramarginal gyrus, predicted greater improvement in depressive symptoms following SSRI treatment. The supramarginal gyrus is part of the FPN, which plays a key role in cognitive flexibility and attentional control [41]. A prior study in adults with depression reported that treatment responders, compared to non-responders, exhibited greater rsFC between the FPN and the precuneus, a core DMN region [24]. The authors suggested that this increased connectivity may reflect the FPN’s capacity to regulate or suppress excessive self-referential processing associated with DMN hyperactivity [24]. Consistent with this, the present finding that stronger rsFC between the core regions of DMN and the supramarginal gyrus predicted better SSRI response may similarly indicate that the FPN contributes to modulating maladaptive internally focused thought processes often seen in depression, thereby supporting more favorable treatment outcomes.

Taken together, these findings suggest that greater baseline rsFC between the DMN and regions in the SMN and FPN may predict better response to escitalopram in medication-naïve adolescents with MDD, who are otherwise healthy and lack significant comorbidity. This supports the potential utility of DMN rsFC patterns as biomarkers for antidepressant treatment outcomes in adolescent depression. However, rsFCs within the DMN were not associated with the prediction of SSRI treatment response. This finding diverges in one respect from that of a previous study, which reported that lower within-DMN rsFC, particularly within the medial temporal lobe DMN subsystem involving the hippocampus and adjacent cortices, predicts better treatment outcomes [29]. Unlike the previous study, the present study focused on the DMN core system, including the vMPFC, dMPFC, and PCC. The hippocampus has been implicated in self-related memory [46], whereas the midline core of the DMN is more closely associated with self-referential processing [26, 50]. These functional distinctions may account for the discrepant findings.

This study has some limitations. The use of an open-label design limits the ability to distinguish true pharmacological responders from those showing placebo-like effects. Treatment response was assessed only for escitalopram, which may restrict the generalizability of findings to other SSRIs. Factors such as trauma or abuse history that reportedly influence SSRI response were not accounted for. Although seed-based rsFC analysis enables targeted investigation of DMN connectivity, it does not capture effects in other potentially relevant large-scale networks. The sample consisted of medication-naïve adolescents with MDD, which may limit generalizability to broader clinical populations. Although medication adherence was monitored using pill counts, this method may not fully ensure actual medication intake, and the possibility of non-adherence cannot be entirely excluded. Finally, because resting-state fMRI relies on unconstrained mental activity, variability in internal states such as drowsiness, mind wandering, or ruminative thinking during scanning cannot be completely ruled out.

In conclusion, our findings suggest that baseline DMN rsFC may predict treatment response to escitalopram in adolescents with depression, as indexed by change in CDRS-R score. Specifically, adolescents with stronger vMPFC-postcentral gyrus, vMPFC-insula, dMPFC-supramarginal gyrus and PCC-supramarginal gyrus connectivity at baseline showed more favorable outcomes. These results support the potential of DMN rsFC as a neural biomarker for predicting SSRI treatment response in adolescent MDD. Identifying such biomarkers is essential for advancing precision psychiatry, enabling clinicians to bypass ineffective treatments in likely non-responders and rapidly implement more personalized, evidence-based interventions. Future studies using larger samples and predictive modeling approaches are needed to evaluate classification performance and determine clinically meaningful thresholds for treatment decision-making.

## Supplementary information

Supplementary material

## Data Availability

Deidentified clinical and rsfMRI summary data are available from the corresponding author upon reasonable request, subject to IRB approval and a data use agreement.
