# Supplementary material for: Resting-state functional connectivity of the default mode network as a predictor for escitalopram response in adolescents with depression
Source: Neuropsychopharmacology. 2026 May 21;51(11):2037–44. doi: 10.1038/s41386-026-02442-x (PMC13597506; doi:10.1038/s41386-026-02442-x)
Supplement: Supplementary file 1 [file 41386_2026_2442_MOESM1_ESM.docx]

**Resting-state Functional Connectivity of Default Mode Network**

**as a Predictor for Escitalopram Response**

**in Adolescents with Depression**

Moonyoung Jang, MD, PhD^1,2*^, Kyung Hwa Lee, PhD^2,†^, Jiyoon Shin, MD, PhD^3^, Jung Lee, MD^4^, Jae Hyun Yoo, MD, PhD^5^, Jeeyoung Chun, MD, PhD^2^, Jisung Ahn, MD^2^, Jae-Won Kim, MD, PhD^2, †^

^1^Department of Psychiatry, Korea University Guro Hospital, Seoul, South Korea

^2^Department of Psychiatry, Seoul National University Hospital and Seoul National University College of Medicine, Seoul, South Korea

^3^Department of Psychiatry, Boramae Medical Center, Seoul, South Korea

^4^Integrative Care Hub, Seoul National University Children’s Hospital, Seoul, South Korea

^5^Department of Psychiatry, The Catholic University of Korea, Seoul St Mary’s Hospital, Seoul, South Korea

^†^J-W K and K H L contributed equally to this work as corresponding authors

**Address for Correspondence**

Professor Jae-Won Kim, M.D., Ph.D.

Department of Psychiatry, Seoul National University Hospital, Seoul, South Korea

101 Daehak-no, Jongno-gu, Seoul, Republic of Korea, 03080

Tel.: +82 2 2072 3648

Fax: +82 2 747 2471

E-mail: [kimjw412@snu.ac.kr](mailto:kimjw412@snu.ac.kr)

Professor Kyung Hwa Lee, Ph.D.

Department of Psychiatry, Seoul National University Hospital, Seoul, South Korea

101 Daehak-no, Jongno-gu, Seoul, Republic of Korea, 03080

Tel.: +82 2 2072 3648

E-mail: [kyunghwalee365@gmail.com](mailto:kyunghwalee365@gmail.com)

**Supplementary Methods**

**Participants**

A total of 95 adolescents with MDD were included in the study. Participants were recruited from both inpatient and outpatient services at the Department of Child and Adolescent Psychiatry, Seoul National University Hospital.

Inclusion criteria consisted of adolescents aged 12 to 17 years who met the Diagnostic and Statistical Manual of Mental Disorders, Fifth Edition (DSM-5) [1] criteria for MDD. Participants were initially identified during routine clinical evaluations by a board-certified child and adolescent psychiatrist (J.-W.K.) who suspected MDD based on clinical assessment. Diagnosis was established using the Kiddie Schedule for Affective Disorders and Schizophrenia for School-Age Children-Present and Lifetime Version (K-SADS-PL) [2], administered by trained clinical psychologists. All interviewers were required to achieve at least 80% agreement with a certified rater prior to conducting independent assessments, and inter-rater reliability for psychiatric diagnoses was high (κ ≥ 0.80). Final diagnostic confirmation was made by the study psychiatrist (J.-W.K.) based on DSM-5 criteria prior to study enrollment. All diagnostic assessments were completed prior to MRI acquisition, and resting-state fMRI scanning was conducted before the initiation of SSRI treatment.

Exclusion criteria were: (a) chronic medical conditions; (b) a history of psychotic disorders, including schizophrenia or bipolar disorder; (c) a history of eating disorders; (d) developmental disorders such as autism spectrum disorder; (e) alcohol or other substance use disorder within the past 6 months; (f) neurological or physical illness; (g) a first-degree relative with bipolar I disorder; and (h) current or prior use of psychiatric medications. Adolescents with a first-degree relative with bipolar I disorder were excluded to minimize the inclusion of individuals with a high familial risk for bipolar spectrum disorders [3, 4], which may influence antidepressant response and increase the risk of antidepressant-induced manic switching.

All adolescents included in the study were medication-naïve, and approximately 80% were experiencing their first depressive episode at the time of enrollment, whereas the remaining participants had a history of at least one prior depressive episode. Information regarding prior psychotherapy or other non-pharmacological mental health services before enrollment was not systematically assessed as part of the study protocol. Comorbid psychiatric diagnoses, including anxiety disorders, attention-deficit/hyperactivity disorder (ADHD), disruptive behavior disorders, tic disorders, and post-traumatic stress disorder (PTSD), were assessed using the K-SADS-PL. Suicidality was assessed using the Columbia-Suicide Severity Rating Scale (C-SSRS). These conditions were not exclusionary unless immediate clinical intervention was required. The prevalence of comorbid diagnoses and suicidality at baseline is summarized in Supplementary Table 1.

Of the 95 adolescents initially enrolled, participants were subsequently excluded due to excessive head motion, imaging artifacts, or anatomical abnormalities identified during preprocessing (Supplementary Figure 1). The demographic and clinical characteristics of the final analytic sample included in the imaging analyses are summarized in Table 1. Correlations between demographic variables and clinical measures are presented in Supplementary Table 2.

The study was approved by the Institutional Review Board at Seoul National University Hospital (IRB no. 1504-113-668). Written informed consent was obtained from both participants and their legal guardians following a full explanation of study procedures. This study adhered to the Strengthening the Reporting of Observational Studies in Epidemiology (STROBE) guidelines for cohort studies.

**SSRI Treatment Procedures**

All adolescents with MDD participated in an 8-week open-label trial of escitalopram [1, 5]. The initial dosage was 5 mg/day during the first week, increased to 10 mg/day beginning in the second week. Dosage adjustments were made at weeks 4 and 6 based on clinical judgment. Escitalopram could be increased in 5 or 10 mg increments or decreased in 5 mg decrements, with a maximum allowable dose of 30 mg/day. Final dosing was individualized to achieve therapeutic efficacy, as determined by the investigator (J.-W.K.) based on symptom improvement and adverse effect monitoring. Once an optimal dose was established, it was maintained through week 8. The final escitalopram dose maintained through week 8 (or at early discontinuation) was used for descriptive analyses in Supplementary Table 3. Concurrent psychotherapy, including cognitive behavioral therapy, was not permitted during the trial. Treatment adherence was assessed through pill counts [2]. Participants were withdrawn from the study if they were noncompliant, defined as consuming less than 60% of prescribed doses at two consecutive visits.

**Depressive Symptom Assessment**

Depressive symptom severity was measured using the Children’s Depression Rating Scale-Revised (CDRS-R) [6], a clinician-rated instrument widely used to assess depression and treatment response in pediatric and adolescent populations, including individuals up to age 17. The CDRS-R has been used extensively in clinical trials of antidepressant treatment in adolescents and has demonstrated good reliability and validity in this age group [7, 8]. The CDRS-R was administered by trained psychologists blinded to participants’ self-report measures at each assessment. The rating was based on a semi-structured interview with the participant, supplemented by information obtained from the parent or caregiver and by behavioral observations made during the interview, consistent with standard administration procedures. In this study, interrater reliability was high, with an intra-class correlation coefficient of 0.96 between two independent raters. Adolescents attended clinical visits at weeks 1, 2, 4, 6, and 8 during SSRI treatment, and the CDRS-R was administered at each visit. The primary outcome was treatment response, defined as the change in CDRS-R score from baseline (week 0) to either week 8 or the point of early study discontinuation. Greater reductions in CDRS-R score indicated greater treatment response. Among participants included in the final analytic sample (n = 70), five participants discontinued the study prior to week 8 but had usable baseline MRI data and at least one post-baseline clinical assessment. Specifically, two participants discontinued after the week-2 visit, two after the week-4 visit, and one after the week-6 visit. All early discontinuations were due to loss to follow-up. For these participants, treatment response was calculated using the last available CDRS-R score obtained prior to discontinuation, consistent with the predefined outcome definition. Adjusted CDRS-R total scores were used for all statistical analyses [9].

**Sensitivity and Additional analyses**

***Associations Between Baseline rsFC and Escitalopram Dose***

To examine whether medication dosing was associated with baseline functional connectivity, additional linear regression analyses were conducted with final escitalopram dose as the dependent variable and each baseline rsFC measure as the predictor. Age and sex were included as covariates. In addition, a separate regression analysis was conducted to examine whether final escitalopram dose was associated with treatment response (change in CDRS-R score), adjusting for age and sex.

***Sensitivity Analyses Addressing Baseline Symptom Severity***

To evaluate the potential influence of baseline symptom severity on the observed associations, we conducted complementary sensitivity analyses using two approaches. First, percent symptom change was used as the dependent variable, calculated as (CDRS-R change / baseline CDRS-R) × 100. Second, we repeated the regression models including baseline CDRS-R total score as an additional covariate, together with age and sex. All models were performed using the extracted seed-to-cluster rsFC values corresponding to the significant voxel-wise findings.

***Sensitivity Analysis Controlling for Anxiety Comorbidity***

To examine whether comorbid anxiety disorders influenced the observed associations, additional regression analyses were conducted including anxiety disorder comorbidity as a covariate along with age and sex.

***Episode Recurrence-Related Analyses***

To examine the potential influence of illness recurrence, additional exploratory analyses were conducted comparing participants with a first depressive episode and those with a history of previous episodes. Group differences in treatment response (CDRS-R change and percent symptom change) and baseline rsFC values of significant clusters identified in the primary analyses were assessed.

***Sensitivity Analysis Controlling for Head Motion***

To examine whether head motion influenced the observed associations, additional regression analyses were conducted including mean head motion as a covariate along with age and sex.

***Sensitivity Analysis Using Global Signal Regression (GSR)***

We evaluated whether the main findings were robust to the application of global signal regression (GSR). To do this, we re-analyzed our rsfMRI data by applying GSR during the preprocessing. Then we re-conducted the 1^st^ level analysis for all participants. To examine whether our results hold when applying global signal regression, we extracted z-transformed rsFC between the DMN seeds and clusters (e.g., vMPFC-postcentral, vMPFC-insula, dMPFC-supramarginal, and PCC-supramarginal) identified the original analysis (without GSR). Multiple linear regression analyses were then performed with change in CDRS-R score as the dependent variable and the rsFC values extracted functional maps applying GSR as the predictor, controlling for age and sex.

***Sensitivity Analysis Using an Adolescent-Specific Template***

Given that our participants were adolescents whose brain is still developing, we tried to use the adolescent-specific template when normalizing rsfMRI data. This may allow us to examine whether our main results were influenced by the use of an adult standard template during spatial normalization. We have normalized our rsfMRI data using the MNIPediatricAsym, cohort 6; age range 13–18.5 years [10]. The normalized data with MNIPediatricAsym template were smoothed and further processed in the same way as we originally analyzed. We re-performed the 1^st^ level analysis using the same DMN seeds and voxel-wise whole brain analyses for each seed. For clusters identified in the adolescent-template analysis, rsFC values between each seed and the corresponding cluster were extracted, and multiple linear regression analyses were performed with change in CDRS-R score as the dependent variable and rsFC as the predictor, controlling for age and sex.

**Supplementary Results**

**Demographic and clinical characteristics**

Of the 95 adolescents with MDD initially recruited, 2 withdrew their consent after screening, 1 improved depressive symptom after screening, four were lost to follow-up, and 18 were excluded due to either anatomical abnormalities on MRI or excessive imaging artifacts. Therefore, 70 participants were included in the final analysis. Among these, five participants discontinued SSRI treatment prior to week 8 due to loss to follow-up but had usable baseline MRI data and at least one post-baseline CDRS-R assessment. Specifically, two participants discontinued after the week-2 visit, two after the week-4 visit, and one after the week-6 visit. These participants were retained in the analyses using their last available CDRS-R score to define treatment response. The flow of participants through the study is illustrated in Supplementary Figure 1.

Participants included in the imaging analyses did not differ significantly from those excluded in sex distribution, IQ, baseline depression severity, treatment response, or escitalopram dose. However, excluded participants were slightly younger than those included in the final analytic sample. Detailed comparisons are presented in Supplementary Table 4. The mean percentage of censored frames was 2.52% (SD = 3.46) and was not significantly associated with age, IQ, baseline CDRS-R score, symptom change, or final escitalopram dose (all *p_s_* > .35). A modest association with sex was observed (*r* = -0.242, *p* = .044; Supplementary Table 5).

**Prediction of treatment response to escitalopram using baseline DMN rsFC**

This study evaluated whether baseline DMN rsFC could predict response to escitalopram over 8 weeks.

Participants’ age, sex, IQ, CDRS-R scores (baseline, post-treatment, and change), and daily escitalopram dose are summarized in Table 1. Categorical treatment response rates based on conventional CDRS-R criteria [9] are presented in Supplementary Table 6. The trajectory of depressive symptom change across treatment visits is illustrated in Supplementary Figure 3, which shows the mean CDRS-R scores at each clinical assessment with standard error bars.

**Supplementary Figures**

**Supplementary Figure 1**. Participant flow diagram. Flow of participants from study enrollment through imaging quality control and clinical outcome assessment.

**Supplementary Figure 2.** Seed regions of interest (ROIs) within the default mode network (DMN) used in resting-state functional connectivity (rsFC) analyses. Each spherical ROI had a 10-mm radius and was centered at MNI coordinates identified in a prior meta-analysis of the DMN. (A) Ventromedial prefrontal cortex (vMPFC; *x* = 0, *y* = 26, *z* = –18). (B) Dorsomedial prefrontal cortex (dMPFC; *x* = 0, *y* = 52, *z* = 26). (C) Posterior cingulate cortex (PCC; *x* = –8, *y* = –56, *z* = 26). All seed regions are displayed on a standard anatomical template in MNI space.

**Supplementary Figure 3**. Trajectory of depressive symptoms during escitalopram treatment. Mean Children’s Depression Rating Scale–Revised (CDRS-R) scores across clinical visits (baseline, weeks 1, 2, 4, 6, and 8) during the 8-week escitalopram treatment period. Error bars represent the standard error of the mean (SEM). CDRS-R scores showed a progressive reduction over the course of treatment.

**Supplementary Figure 4**. Partial regression plots illustrating the association between baseline rsFC and change in CDRS-R score using functional connectivity values derived from data preprocessed with global signal regression. (A) vMPFC–postcentral gyrus, (B) vMPFC–insula, (C) dMPFC–supramarginal gyrus, (D) PCC–supramarginal gyrus. All regression models controlled for age and sex.

**Supplementary Figure 5**. Adolescent-template analysis for vMPFC–left postcentral connectivity. (A) Cluster identified in the voxel-wise regression analysis using the adolescent-specific template (MNIPediatricAsym, cohort 6), showing baseline functional connectivity between the ventromedial prefrontal cortex (vMPFC) seed and the left postcentral gyrus associated with treatment response. (B) Partial regression plot illustrating the association between baseline vMPFC–left postcentral rsFC and change in Children’s Depression Rating Scale–Revised (CDRS-R) score after controlling for age and sex. Stronger baseline connectivity was associated with greater reductions in depressive symptoms.

**Supplementary Figure 6**. Adolescent-template analysis for vMPFC–left posterior insula connectivity. (A) Cluster identified in the voxel-wise regression analysis using the adolescent-specific template (MNIPediatricAsym, cohort 6), showing baseline functional connectivity between the ventromedial prefrontal cortex (vMPFC) seed and the left posterior insula associated with treatment response. (B) Partial regression plot illustrating the association between baseline vMPFC–left posterior insula rsFC and change in Children’s Depression Rating Scale–Revised (CDRS-R) score after controlling for age and sex. Stronger baseline connectivity was associated with greater reductions in depressive symptoms.

**Supplementary Figure 7**. Adolescent-template analysis for dMPFC–right supramarginal connectivity. (A) Cluster identified in the voxel-wise regression analysis using the adolescent-specific template (MNIPediatricAsym, cohort 6), showing baseline functional connectivity between the dorsomedial prefrontal cortex (dMPFC) seed and the right supramarginal gyrus associated with treatment response. (B) Partial regression plot illustrating the association between baseline dMPFC–right supramarginal rsFC and change in Children’s Depression Rating Scale–Revised (CDRS-R) score after controlling for age and sex. Stronger baseline connectivity was associated with greater reductions in depressive symptoms.

**Supplementary Figure 8**. Adolescent-template analysis for PCC–right supramarginal connectivity. (A) Cluster identified in the voxel-wise regression analysis using the adolescent-specific template (MNIPediatricAsym, cohort 6), showing baseline functional connectivity between the posterior cingulate cortex (PCC) seed and the right supramarginal gyrus associated with treatment response. (B) Partial regression plot illustrating the association between baseline PCC–right supramarginal rsFC and change in Children’s Depression Rating Scale–Revised (CDRS-R) score after controlling for age and sex. Stronger baseline connectivity was associated with greater reductions in depressive symptoms.

**Supplementary Tables**

| Supplementary Table 1. Distribution of comorbid diagnoses at baseline | | |
| --- | --- | --- |
| Variable | | N (%) |
| Comorbid diagnoses | |  |
|  | Any comorbidity | 40 (57.1%) |
|  | Anxiety disorders | 32 (45.7%) |
|  | ADHD | 1 (1.4%) |
|  | Disruptive behavior disorders (ODD/CD) | 8 (11.4%) |
|  | PTSD | 6 (8.6%) |
|  | Tic disorders | 7 (10.0%) |
| Suicidality | |  |
|  | Suicidal ideation | 51 (72.9%) |
|  | Suicide attempt history | 19 (27.1%) |
| Note. ODD = oppositional defiant disorder; CD = conduct disorder.  Comorbid diagnoses assessed suing the K-SADS-PL. Suicidality were assessed using the Columbia-Suicide Severity Rating Scale. | | |

| Supplementary Table 2. Correlations between demographic variables and CDRS-R clinical measures. | | | |  |
| --- | --- | --- | --- | --- |
| Variable | Baseline CDRS | Follow-up CDRS | CDRS change | % CDRS change |
| Age | *r* = 0.204, *p* = .090 | *r* = 0.042, *p* = .730 | *r* = -0.124, *p* = .305 | *r* = -0.072, *p* = .555 |
| Sex | *r* = -0.044, *p* = .717 | *r* = 0.055, *p* = .653 | *r* = 0.083, *p* = .496 | *r* = 0.045, *p* = .712 |
| IQ | *r* = -0.134, *p* = .269 | *r* = -0.037, *p* = .760 | *r* = 0.073, *p* = .547 | *r* = 0.082, *p* = .500 |
| Mean head motion | *r* = -0.087, *p* = .476 | *r* = 0.044, *p* = .716 | *r* = 0.107, *p* = .377 | *r* = 0.089, *p* = .461 |
| Note: Values represent Pearson correlations. CDRS-R (Children’s Depression Rating Scale–Revised) change was calculated as the difference between the final available CDRS-R score and the baseline score. Percent symptom change was calculated as (CDRS-R change / baseline CDRS-R) × 100. Follow-up CDRS-R refers to the final available assessment (week 8 or the last visit prior to early discontinuation). Head motion was calculated as the Euclidean distance between the six rigid-body head motion parameters at two consecutive time points. | | | | |
|  |  |  |  |  |
|  |  |  |  |  |
|  |  |  |  |  |

| Supplementary Table 3. Distribution of final escitalopram doses in the final analytic sample (N = 70) | |
| --- | --- |
| Final escitalopram dose, mg/day | N (%) |
| 5 | 5 (7.1) |
| 10 | 10 (14.3) |
| 15 | 11 (15.7) |
| 20 | 29 (41.4) |
| 25 | 14 (20.0) |
| 30 | 1 (1.4) |

| Supplementary Table 4. Demographic and Clinical Characteristics of Included and Excluded Participants | | | | |
| --- | --- | --- | --- | --- |
| Variable | | Included (n = 70) | Excluded (n = 25) | *p*-value |
| Demographic | |  |  |  |
|  | Age, mean ± SD | 15.09 ± 1.48 | 13.82 ± 1.62 | < 0.001 |
|  | Female, n (%) | 49 (70.0%) | 16 (64.0%) | 0.580 |
| Cognitive | |  |  |  |
|  | IQ, mean ± SD | 104.89 ± 14.37 | 106.18 ± 13.19 | 0.708 |
| Clinical | |  |  |  |
|  | Any comorbidity, n (%) | 40 (57.1%) | 15 (60.0%) | 1.000 |
|  | ADHD comorbidity, n (%) | 1 (1.4%) | 2 (8.0%) | 0.168 |
|  | Baseline CDRS-R, mean ± SD | 58.69 ± 11.11 | 62.50 ± 13.41 | 0.185 |
|  | CDRS-R change, mean ± SD | -15.19 ± 14.07 | -17.71 ± 12.71 | 0.463 |
|  | Final escitalopram dose (mg), mean ± SD | 17.93 ± 5.94 | 17.61 ± 6.19 | 0.825 |

| Supplementary Table 5. Percentage of censored volumes due to head motion or artifact and its associations with clinical variables | | |
| --- | --- | --- |
|  |  |  |
| Variable | *r* | *p* |
| Age | -0.113 | .353 |
| IQ | 0.067 | .581 |
| Baseline CDRS-R | 0.075 | .535 |
| CDRS-R change | 0.108 | .371 |
| Final escitalopram dose | 0.072 | .554 |
| Sex | -0.242 | .044 |

| Supplementary Table 6. Categorical Treatment Response and Remission Based on CDRS-R Criteria (N = 70) | |
| --- | --- |
| Outcome definition | N (%) |
| reduction of 40% or greater from baseline | 33 (47.1%) |
| Remission (CDRS-R ≤ 28) | 6 (8.6%) |
| Note. Treatment response was defined as a 40% or greater reduction in CDRS-R total score from baseline to week 8 (or the last available assessment in cases of early discontinuation). Remission was defined as a CDRS-R total score of 28 or lower at endpoint. The primary analyses in the main manuscript used continuous change in CDRS-R score as the outcome measure. | |
|  |  |
|  |  |

| Supplementary Table 7. Associations between final escitalopram dose and baseline rsFC | | | |
| --- | --- | --- | --- |
| Predictor | *β* | *t* | *p* |
| vMPFC–postcentral gyrus | 0.0025 | 1.1 | 0.276 |
| vMPFC–posterior insula | 0.0013 | 0.69 | 0.493 |
| dMPFC–supramarginal gyrus | 0.0036 | 0.92 | 0.359 |
| PCC–supramarginal gyrus | 0.0051 | 1.74 | 0.087 |
| Note. rsFC = resting-state functional connectivity; vMPFC = ventromedial prefrontal cortex; dMPFC = dorsomedial prefrontal cortex; PCC = posterior cingulate cortex. | | | |
|  |  |  |  |

| Supplementary Table 8. Sensitivity Analyses Addressing Baseline Symptom Severity | |  |  |  |
| --- | --- | --- | --- | --- |
| Model | Predictor | *β* | *t* | *p* |
| Percent change (CDRS-R change / baseline CDRS-R) × 100 | vMPFC–insula rsFC | -0.62 | -6.3 | <.001 |
|  | vMPFC–postcentral rsFC | -0.58 | -5.72 | <.001 |
|  | PCC–supramarginal rsFC | -0.52 | -4.96 | <.001 |
|  | dMPFC–supramarginal rsFC | -0.46 | -4.15 | <.001 |
| Baseline-adjusted model (change score + baseline CDRS covariate) | vMPFC–insula rsFC | -0.5 | -5.69 | <.001 |
|  | vMPFC–postcentral rsFC | -0.46 | -4.94 | <.001 |
|  | PCC–supramarginal rsFC | -0.44 | -4.75 | <.001 |
|  | dMPFC–supramarginal rsFC | -0.37 | -3.67 | <.001 |
| Note. Percent change was calculated as (CDRS-R change / baseline CDRS-R) × 100. Baseline-adjusted models included baseline CDRS-R score as a covariate. *β* values represent standardized regression coefficients. All models included age and sex as covariates. | | | | |
|  |  |  |  |  |

| Supplementary Table 9. Sensitivity analyses controlling for anxiety disorder comorbidity | | | | |  |  |
| --- | --- | --- | --- | --- | --- | --- |
| Predictor | *β* | SE | *t* | *p* | Anxiety comorbidity *β* | Anxiety comorbidity *p* |
| vMPFC–postcentral rsFC | -0.579 | 0.101 | -5.736 | < .001 | -0.081 | .423 |
| vMPFC–insula rsFC | -0.637 | 0.098 | -6.510 | < .001 | -0.179 | .073 |
| dMPFC–supramarginal rsFC | -0.498 | 0.110 | -4.537 | < .001 | 0.001 | .990 |
| PCC–supramarginal rsFC | -0.543 | 0.103 | -5.26 | < .001 | -0.077 | .459 |
| Note. *β* values represent standardized regression coefficients. Each regression model included age, sex, and anxiety disorder comorbidity as covariates. | | | | | | |

| Supplementary Table 10. Sensitivity analyses, controlling for mean head motion | | | |  |  |  |
| --- | --- | --- | --- | --- | --- | --- |
| Predictor | *β* | SE | *t* | *p* | Mean head motion *β* | Mean head motion *p* |
| vMPFC–postcentral rsFC | -0.569 | 0.101 | -5.636 | < .001 | 0.100 | 0.339 |
| vMPFC–insula rsFC | -0.596 | 0.098 | -6.055 | < .001 | 0.087 | 0.397 |
| dMPFC–supramarginal rsFC | -0.489 | 0.109 | -4.490 | < .001 | 0.094 | 0.402 |
| PCC–supramarginal rsFC | -0.531 | 0.104 | -5.121 | < .001 | 0.081 | 0.453 |
| Note. *β* values represent standardized regression coefficients. Each regression model included age, sex, and mean head motion as covariates. Mean head motion *β* and *p* are reported to show the effect of mean head motion on the prediction. | | | | | | |

| Supplementary Table 11. Sensitivity analyses using global signal regression | | |  |  |
| --- | --- | --- | --- | --- |
|  | non-GSR rsFC | | GSR-processed rsFC | |
| Connectivity | Partial *r* | *p*-value | Partial *r* | *p*-value |
| vMPFC–postcentral | −0.576 | < .001 | −0.304 | .012 |
| vMPFC–insula | −0.603 | < .001 | −0.449 | < .001 |
| dMPFC–supramarginal | −0.498 | < .001 | −0.379 | .001 |
| PCC–supramarginal | −0.540 | < .001 | −0.438 | < .001 |
| Note. Partial *r* values represent partial correlations between rsFC and change in CDRS-R score derived from multiple linear regression models controlling for age and sex. The first set of analyses used rsFC values derived from preprocessing including global signal regression (GSR), whereas the second set reflects the primary analyses performed without GSR. regression models predicting change in CDRS-R score. Each model included age and sex as covariates. | | | | |
|  |  |  |  |  |
|  |  |  |  |  |

**References**

1.	Lee KH, Shin J, Lee J, Yoo JH, Kim JW, Brent DA. Measures of Connectivity and Dorsolateral Prefrontal Cortex Volumes and Depressive Symptoms Following Treatment With Selective Serotonin Reuptake Inhibitors in Adolescents. JAMA Netw Open. 2023;6(8):e2327331.

2.	Woldu H, Porta G, Goldstein T, Sakolsky D, Perel J, Emslie G, et al. Pharmacokinetically and clinician-determined adherence to an antidepressant regimen and clinical outcome in the TORDIA trial. J Am Acad Child Adolesc Psychiatry. 2011;50(5):490-8.

3.	Goldberg JF, Truman CJ. Antidepressant-induced mania: an overview of current controversies. Bipolar Disord. 2003;5(6):407-20.

4.	Baumer FM, Howe M, Gallelli K, Simeonova DI, Hallmayer J, Chang KD. A pilot study of antidepressant-induced mania in pediatric bipolar disorder: Characteristics, risk factors, and the serotonin transporter gene. Biol Psychiatry. 2006;60(9):1005-12.

5.	Lee J, Lee KH, Kim SH, Han JY, Hong SB, Cho SC, et al. Early changes of serum BDNF and SSRI response in adolescents with major depressive disorder. J Affect Disord. 2020;265:325-32.

6.	Poznanski EO, Mokros HB, Western Psychological S. Children's depression rating scale, revised (CDRS-R). Los Angeles, Calif.: Western Psychological Services; 1996.

7.	March JS, Silva S, Petrycki S, Curry J, Wells K, Fairbank J, et al. The Treatment for Adolescents With Depression Study (TADS): long-term effectiveness and safety outcomes. Arch Gen Psychiatry. 2007;64(10):1132-43.

8.	Emslie GJ, Heiligenstein JH, Wagner KD, Hoog SL, Ernest DE, Brown E, et al. Fluoxetine for acute treatment of depression in children and adolescents: a placebo-controlled, randomized clinical trial. J Am Acad Child Adolesc Psychiatry. 2002;41(10):1205-15.

9.	Wagner KD, Ambrosini P, Rynn M, Wohlberg C, Yang R, Greenbaum MS, et al. Efficacy of sertraline in the treatment of children and adolescents with major depressive disorder: two randomized controlled trials. Jama. 2003;290(8):1033-41.

10.	Fonov V, Evans AC, Botteron K, Almli CR, McKinstry RC, Collins DL. Unbiased average age-appropriate atlases for pediatric studies. Neuroimage. 2011;54(1):313-27.
